# Changes in the Fecal Metabolome Accompany an Increase in Aberrant Crypt Foci in the Colon of C57BL/6 Mice Fed with a High-Fat Diet

**DOI:** 10.3390/biomedicines10112891

**Published:** 2022-11-11

**Authors:** Huawei Zeng, Bryan D. Safratowich, Wen-Hsing Cheng, Andrew D. Magnuson, Matthew J. Picklo

**Affiliations:** 1 United States Department of Agriculture, Agricultural Research Service, Grand Forks Human Nutrition Research Center, Grand Forks, ND 58203, USA; 2 Department of Food Science, Nutrition and Health Promotion, Mississippi State University, Starkville, MS 39762, USA

**Keywords:** aberrant crypt foci, fatty acids, fecal metabolome, lipid, obesity

## Abstract

High-fat diet (HFD)-induced obesity is a risk factor for colon cancer. Our previous data show that compared to an AIN-93 diet (AIN), a HFD promotes azoxymethane (AOM)-induced colonic aberrant crypt foci (ACF) formation and microbial dysbiosis in C57BL/6 mice. To explore the underlying metabolic basis, we hypothesize that AOM treatment triggers a different fecal metabolomic profile in C57BL/6 mice fed the HFD or the AIN. We found that 65 of 196 identified metabolites were significantly different among the four groups of mice (AIN, AIN + AOM, HFD, and HFD + AOM). A sparse partial least squares discriminant analysis (sPLSDA) showed that concentrations of nine fecal lipid metabolites were increased in the HFD + AOM compared to the HFD, which played a key role in overall metabolome group separation. These nine fecal lipid metabolite concentrations were positively associated with the number of colonic ACF, the cell proliferation of Ki67 proteins, and the abundance of dysbiotic bacteria. These data suggest that the process of AOM-induced ACF formation may increase selective fecal lipid concentrations in mice fed with a HFD but not an AIN. Collectively, the accumulation of these critical fecal lipid species may alter the overall metabolome during tumorigenesis in the colon.

## 1. Introduction

Obesity is one of the most prevalent causes of non-communicable diseases across the globe [1], and there is strong epidemiologic evidence linking diet-induced obesity to increased risk of colon cancer in humans [2,3]. Mechanistic studies in mice show that the consumption of a high-fat diet (HFD) leads to the accumulation of excess body fat similar to those observed in obese humans, namely increased adiposity, chronic inflammation, and risk of cancer in the colon [4]. However, this association may differ by sex and tumor subsites in the colon [5]. For example, obesity is associated with a 30–70% increased risk of colon cancer in men, whereas this association is less consistent and pronounced in women [5,6]. Similarly, male mice are more sensitive to HFD-induced weight gain and colonic inflammation than female mice [7]. As the first step in studying fecal metabolomics, we then focused on determining the impact of AOM treatment on fecal metabolome in male mice in this study, hoping to provide new data for designing future female mouse experiments. We recently reported that in a mouse model, a HFD promotes the formation of azoxymethane (AOM)-induced aberrant crypt foci (ACF) (a putative preneoplastic lesion) and microbial dysbiosis in the colon [8]. However, the underlying metabolic process remains to be determined. 

Metabolic status can be potentially tracked through metabolomics as small-molecule metabolites represent a dynamic situation in response to physiological and pathological changes [9]. Untargeted metabolite profiling may define a molecular phenotype representing the underlying biochemical change [10]. Recent data demonstrate that a HFD drives tumorigenesis in the colon through inducing gut microbial dysbiosis and metabolomic dysregulation [11]. However, the impact of the formation of AOM-induced ACF on fecal metabolome in the colon, when compared to the healthy (untreated) colon, remains largely unknown. As the interplay between cancer cells and tumor microenvironment plays a fundamental role in tumor progression [12], we hypothesize that AOM treatment triggers a different fecal metabolomic profile in C57BL/6 mice fed with the HFD or the AIN-93 diet (AIN) [8]. 

## 2. Results

To determine the impact of the AOM treatment on the fecal metabolome in the colon, we compared the fecal metabolome of the four groups of mice fed with two different diets with and without AOM (AIN, AIN + AOM, HFD, and HFD + AOM) [8].

### 2.1. Effect of AOM Treatment on the Abundance of Fecal Metabolites 

Daily fecal samples were collected and stored at −80 °C during the last week of the study (week 14) [8]. We identified 196 metabolites (Table S1) from 455 discrete signals detected in the fecal samples using GC-TOF-MS, and 65 of 196 identified metabolites were significantly different among the four treatment groups. The relative values for these 65 metabolites compared to the AIN group are shown in Table 1. 

### 2.2. Effect of Diet and AOM Treatment on Overall Fecal Metabolome 

**Figure 1:**
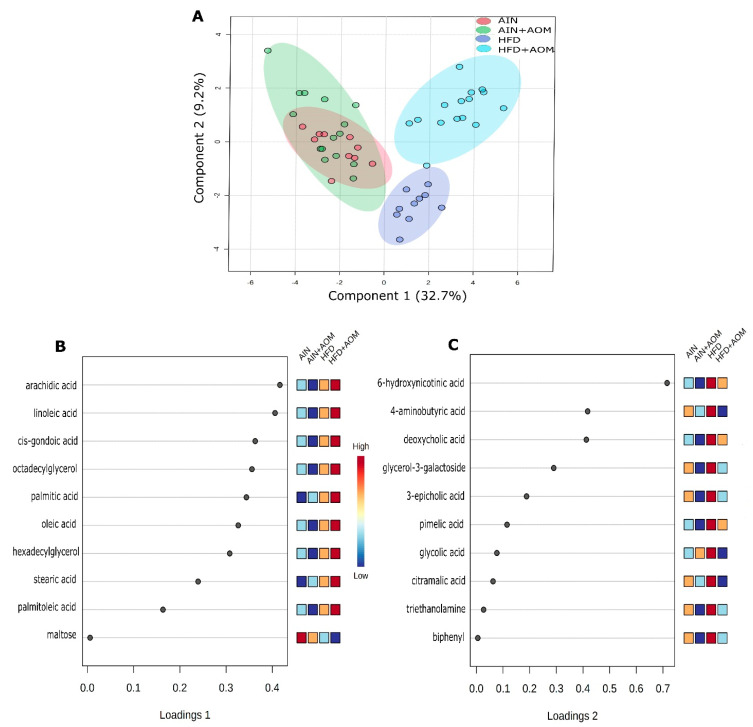
Two-dimensional (2D) sPLS-DA of the 4 experimental groups (**A**) and loading plots of 10 metabolites which are most significant in group separation among the four groups for component 1 (**B**) and component 2 (**C**). Mice without AOM treatment (control), *n* = 10/group (AIN or HFD group). Mice with AOM treatment, *n* = 15/group (AIN + AOM or HFD + AOM group).

To further determine the effect of AOM treatment on the overall metabolome, sparse partial least squares discriminant analysis (sPLSDA) was used to visualize the individual sample variability and overall metabolite separation between experimental groups (Figure 1A). There was separation between the HFD and HFD + AOM groups, but not the AIN and AIN + AOM groups, component 1 (32.7%) played a greater role than that of component 2 (9.2%) for this score plot (Figure 1).

The top 10 metabolites determining component 1 separation were (1) the increased concentrations of arachidic acid (20:0; ≥33%), linoleic acid (18:2n-6; ≥104%), cis-gondoic acid (20:1n-9; ≥192%), octadecylglycerol (≥142%), palmitic acid (16:0; ≥62%), oleic acid (18:1n-9); ≥213%), hexadecylglycerol (≥166%), stearic acid (18:0; ≥29%), and palmitoleic acid (16:1n-7; ≥82%) in the HFD + AOM group relative to other treatments; and (2) the increased concentration of maltose (≥44%) in the AIN group compared to other groups (Figure 1B, Table 1). 

Conversely, the top 10 metabolites in the HFD group for component 2 separation, relative to the HFD + AOM group, were the increased concentrations of 6-hydroxynicotinic acid (≥105%), 4-aminobutyric acid (≥63%), deoxycholic acid (≥144%), glycerol-3-galactoside (≥18%), 3-epicholic acid (≥120%), pimelic acid (≥35%), glycolic acid (≥48%), citramalic acid (≥9%), triethanolamine (≥28%), and biphenyl (≥37%) (Figure 1C, Table 1).

### 2.3. Effect of Diet and AOM Treatment on Metabolic Pathways and Potential Disease Aspects 

**Figure 2:**
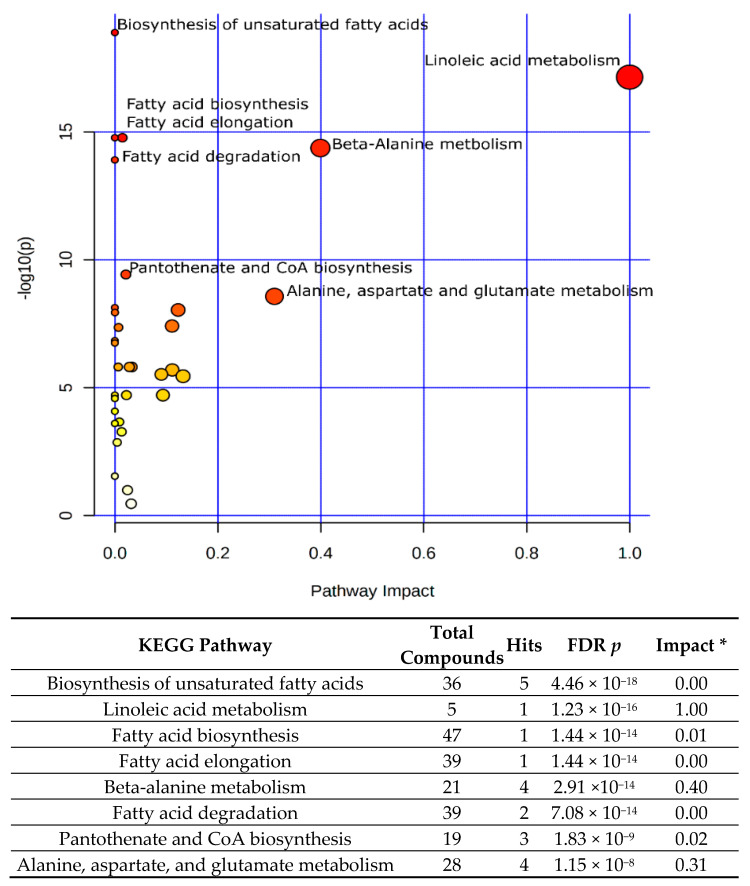
Overall pathway enrichment analysis of the 65 differential metabolites in the AIN, HFD, AIN + AOM, and HFD + AOM groups. Only the top 8 of 32 statistically significant pathways (Table S2) are listed here; the *p*-values are obtained by pathway enrichment analysis and adjusted by both Holm and FDR methods; the *p*-value and red-dot color intensity are inversely proportional. * The pathway impact score is obtained by pathway topology analysis.

These 65 altered metabolites (Table 1) were significantly involved in 32 of 82 biochemical pathways in a pathway library of mice (Table S2). The top 8 significant pathways (Figure 2) included (1) the biosynthesis of unsaturated fatty acids; (2) linoleic acid metabolism; (3) fatty acid biosynthesis; (4) fatty acid elongation; (5) beta-alanine metabolism; (6) fatty acid degradation; (7) pantothenate and CoA biosynthesis; and (8) alanine, aspartate, and glutamate metabolism. 

Although the overall pathway enrichment analysis (Figure 2) demonstrated that fatty acid metabolism was the critical metabolic process in all four experimental groups, it did not address the specific pathways which were most crucial to HFD and ensuing colon cancer. Subsequently, we examined the metabolic pathways of the AIN vs. HFD group (Table 2A) and the HFD vs. HFD + AOM group (Table 2B). Similar to that of the overall pathway analysis (Figure 2), lipid metabolism pathways remained in the top eight pathways (Table 2A,B). However, the significant priorities of the pathways were different between the AIN group vs. the HFD group (Table 2A) and between the HFD group vs. the HFD + AOM group (Table 2B). For example, the most crucial pathway was the biosynthesis of unsaturated fatty acids in the AIN group vs. the HFD group (Table 2A), while there was linoleic acid metabolism in the HFD group vs. the HFD + AOM group (Table 2B). 

**Figure 3:**
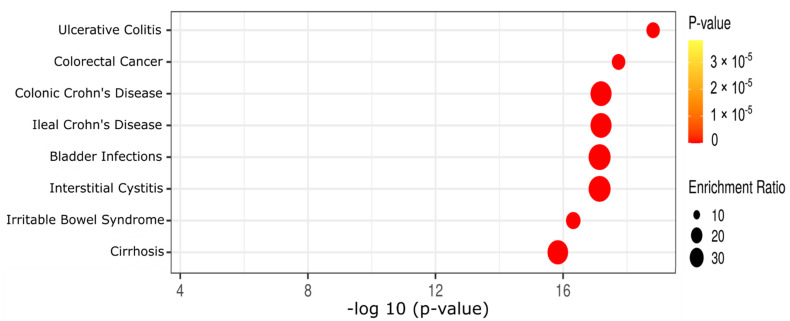
Metabolite set enrichment analysis of the 65 differential metabolites. Only the top 8 of 27 statistically significant metabolite sets (Table S5, with Q-statistic) are listed here, and the *p*-values are obtained by metabolite set enrichment analysis and adjusted by FDR methods.

To further explore the potential roles of these 65 differential metabolites in human disease aspects, we conducted a metabolite set enrichment analysis. These 65 metabolites were significantly involved in 27 of the 44 reported metabolite sets in human feces (Table S5). The top eight reported human fecal metabolite sets (Figure 3, Table S5) included ulcerative colitis, colorectal cancer, colonic Crohn’s diseases, ileal Crohn’s diseases, bladder infections, interstitial cystitis, irritable bowel syndrome, and cirrhosis. 

### 2.4. Correlation between Key Fecal and Plasma Fatty Acids 

**Figure 4:**
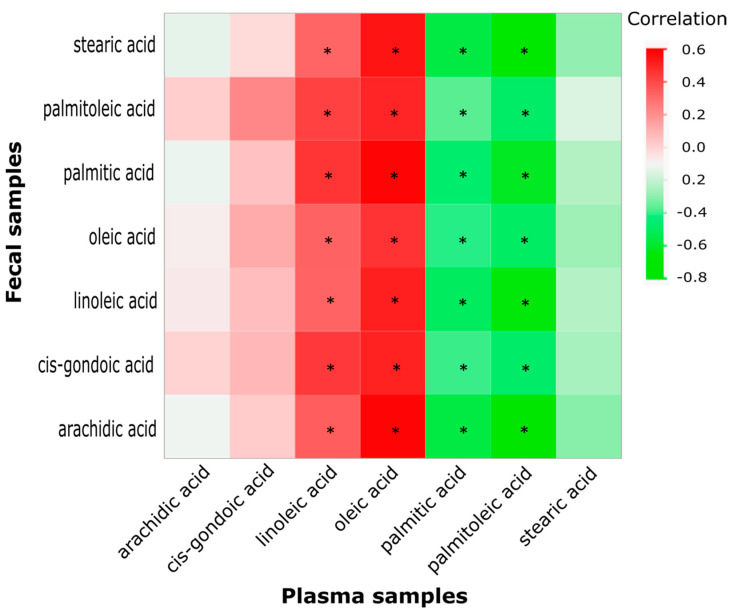
Correlation between the concentrations of key fecal and plasma fatty acids. The heatmap color stands for correlation coefficient r value. * *p* < 0.05, a significant FDR-adjusted correlation (*n* = 50).

As shown in Figure 2 and Figure 3, fatty-acid-related pathways were the top overall metabolic action related to colonic inflammation and cancer. Component 1 (Figure 1B) mainly involved free fatty acids in which all seven fecal fatty acid concentrations were increased in the HFD + AOM group compared to the HFD group. In contrast, our previous study [13] showed that the concentrations of arachidic acid, cis-gondoic acid, oleic acid, palmitic acid, palmitoleic acid, and stearic acid (six of seven of these fatty acids) did not differ, and only the concentration of linoleic acid was increased in the HFD + AOM group compared to the HFD group. To further understand the connection between the colonic microenvironment and body system, we analyzed the correlation (Figure 4) between these seven fecal and plasma fatty acids. The plasma data of seven fatty acids were taken from our previous study [13]. The correlation analysis (Figure 4) showed that linoleic acid and oleic acid in the fecal samples were positively associated with concentrations in the plasma samples, while palmitic acid and palmitoleic acid were inversely associated. However, there was not a correlation between the concentrations of arachidic acid, cis-gondoic acid, and stearic acid in fecal samples and those in plasma samples (Figure 4). 

### 2.5. Correlation between Fecal Metabolites (or Plasma Counterpart Metabolites), AC, ACF, iNOS, Ki67, and Colonic Bacteria 

As shown in Figure 2, Figure 3 and Figure 4, component 1 (Figure 1B) mainly concerned free fatty acids, which might be associated with molecular actions related to colonic inflammation and cancer. In addition to being part of the colonic microenvironment, these fecal fatty acids could potentially be biomarker candidates because they can be non-invasively collected. Therefore, we examined the correlation between component 1 metabolites (Figure 1B) (or the plasma counterpart metabolites) and ACF formation, oncogenic proteins, and bacterial dysbiosis. Data on the plasma, AOM-induced colonic AC, ACF, iNOS, Ki67, Proteobacteria, and Tenericutes were taken from our previous studies [8,13]. 

**Figure 5:**
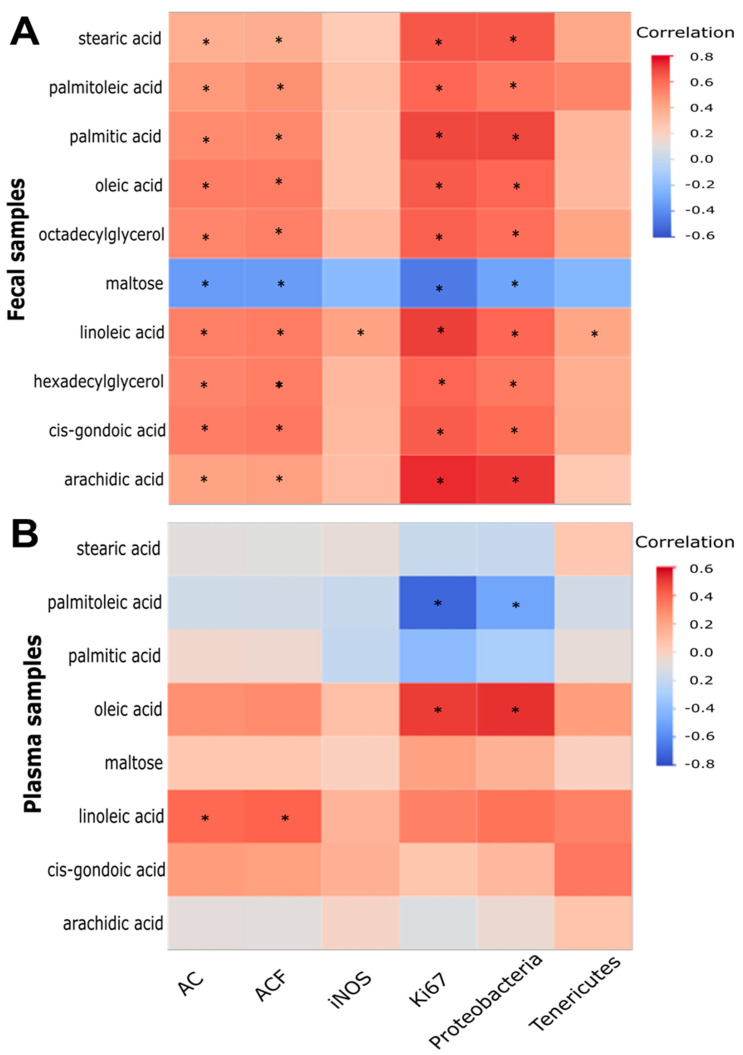
Correlation between the concentrations of panel (**A**)—the 10 metabolites of component 1 (Figure 1) or (**B**)—the 8 plasma counterpart metabolites of component 1 in our previous study [13] **, the number of AC and ACF protein levels of iNOS and Ki67, and the abundance of Proteobacteria and Tenericutes in the ileum/colon in our previous study [8]. The heatmap color stands for a correlation coefficient r value. * *p* < 0.05, a significant FDR-adjusted Spearman correlation (*n* = 44 to 50). ** Octadecylglycerol and hexadecylglycerol could not be detected in the plasma samples in our previous study [13].

Correlation heatmap analysis (Figure 5) revealed the following. (1) Except for an inverse association with maltose, there was a positive association between the concentrations of all nine fecal lipid metabolites of component 1 (Figure 1B); the number of AC, ACF, and Ki67 proteins; and the abundance of proteobacteria in the colon (Figure 5A). Moreover, the concentration of fecal linoleic acid was also positively associated with the iNOS protein and abundance of Tenericutes (Figure 5A). (2) In contrast, when the plasma counterpart metabolites were used in the same correlation analysis (Figure 5B), we found that only the concentrations of plasma linoleic acid and oleic acid were positively associated with (AC and ACF) and (Ki67 and proteobacteria), respectively (Figure 5B). However, the concentration of plasma palmitoleic acid was inversely associated with Ki67 and Proteobacteria (Figure 5B). 

## 3. Discussion

In the present report, 65 of the 196 identified metabolites were significantly different among the four treatment groups (Table 1 and Table S1, Figure 1). Importantly, there was an overall metabolomic separation between the HFD and HFD + AOM groups (Figure 1A), and this observation suggests that AOM-induced ACF formation is associated with a greater fecal metabolomic change in C57BL/6 mice fed with a HFD when compared to an AIN.

The effects of AOM-induced ACF formation on these metabolite concentrations (Table 1) suggest several biological significances. First, it is known that AOM is hydroxylated by the microsomal monooxygenase system in the liver, is immediately conjugated with glucuronic acid, and is transported via the bile to the intestine [14]. The AOM-conjugated glucuronic acid may be hydrolyzed by gut bacterial β-glucuronidase to free methylazoxy methanol, an active carcinogen in the colon [15]. As glucuronic acid is a precursor for p-tolyl glucuronide [15,16], the p-tolyl glucuronide concentration was increased (>100%) in the HFD + AOM group compared to other three groups (Table 1), suggesting a higher level of bacterial β-glucuronidase (a marker for procarcinogenic activity) in the HFD + AOM group [17]. This observation is in line with the idea that a diet high in fat and sucrose and low in calcium and fiber, with a high risk for colon cancer, increases fecal β-glucuronidase activity [17,18]. However, future studies on bacterial glucuronidase activity are warranted to confirm this potential molecular action. 

Metabolites of component 1 were lipid metabolites and their concentrations were greatly increased in the HFD + AOM group compared to the other three groups (Figure 1B). In Figure 1B, 7 of the 10 metabolites of component 1 were fatty acids (arachidic acid, linoleic acid, cis-gondoic acid, palmitic acid, oleic acid, stearic acid, and palmitoleic acid). It has been documented that corn oil contains all of these seven fatty acid species [19] and the HFD in this report was corn-oil-based [8]. Thus, this finding (Figure 1B) suggests that the process of AOM-induced-ACF may be associated with a > one-fold increase in these lipid metabolites in the colon, which may promote and/or exacerbate a procarcinogenic microenvironment for colonic tumorigenesis cancer in the context of HFD consumption. Alternatively, AOM treatment may also impact the absorption of lipids from the gut, a process that only becomes evident in context of the lipid-rich HFD.

Third, except glycolic acid in the AIN group, all 10 endogenous metabolites, such as bile acid byproducts of component 2, were decreased by AOM for both diets (Figure 1C). There are a few probable causes. For example, during the AOM-induced ACF formation, epithelial cell mutations may cause a decrease in a certain nutrient (e.g., cholesterol) uptake [20,21]. Additionally, as there was a weight loss in the AIN + AOM and HFD + AOM groups in our previous data [8], and an intestinal cachexia may occur because of metabolic and nutritional changes during the progression of tumorigenesis [22]. Overall, these abnormal changes in fecal metabolite/fatty acids (Figure 1) correspond to the notion that inflammation caused by the metabolic processing of fatty acids (generated by lipid overdose) increases the risk of tumorigenesis [23]. 

To determine the biological impact of these 65 differential metabolites on the metabolism, pathway enrichment analysis demonstrated that these 65 altered metabolites (Table 1) were significantly involved in 32 biochemical pathways (Table S2). Five of the eight top pathways (among these 32 biochemical pathways) (Figure 2) were related to the biosynthesis, elongation, and degradation of free fatty acids, particularly unsaturated fatty acids. These data are in agreement with the increased concentrations of unsaturated fatty acids, such as linoleic acid, cis-gondoic acid, oleic acid, and palmitoleic acid in the HFD + AOM group (Figure 1B). The linoleic acid metabolism played an important metabolic role because of the high overall impact score (Figure 2). Further pathway dissection analysis showed that the most crucial pathway was the biosynthesis of unsaturated fatty acids in the AIN vs. HFD group (Table 2A), while linoleic acid metabolism in the HFD vs. HFD + AOM group (Table 2B). These mechanistic pathway analyses suggest that these two pathways may be the top metabolic actions specific to the HFD and HFD + AOM treatment, respectively. 

In agreement with the pathway enrichment analysis, metabolite set enrichment analysis also revealed that 65 differential metabolites were statistically matched with 27 metabolite sets (Figure 3, Table S5). Five of the eight top metabolite sets (among those 27 metabolite sets) were involved in gastrointestinal inflammation and diseases such as ulcerative colitis, colorectal cancer, colonic/ileal Crohn’s diseases, and irritable bowel syndrome (Figure 3). To examine the connection between fecal and plasma fatty acids, correlation analysis (Figure 4) was used to show that only the concentrations of linoleic acid and oleic acid in the fecal samples were positively associated with concentrations in the plasma samples. These findings are in line with recent reports that dietary linoleic acid and oleic acid are unsaturated fatty acids known to cause inflammation and promote the development of colon cancer [24,25,26,27].

While the underlying mechanism of fecal lipid accumulation (Figure 1B) remains to be determined, it can be shown that the AOM-induced ACF process may selectively enrich certain bacterial taxa in the colon, which are likely to play a role in fatty acid biosynthesis. For examples, our previous report [8] showed that, at the genus level, the highest increased bacterial taxonomy was lactobacillus due to the HFD + AOM treatment, in which the abundance of colonic lactobacillus in the HFD + AOM group was 3.32% compared to <0.85% in the AIN, AIN + AOM, or HFD groups. It is known that lactobacillus bacteria contain novel multi-component enzyme machinery (e.g., linoleic acid isomerase) which catalyzes C=C double-bond migration for conjugated fatty acid synthesis [28,29]. 

**Figure 6:**
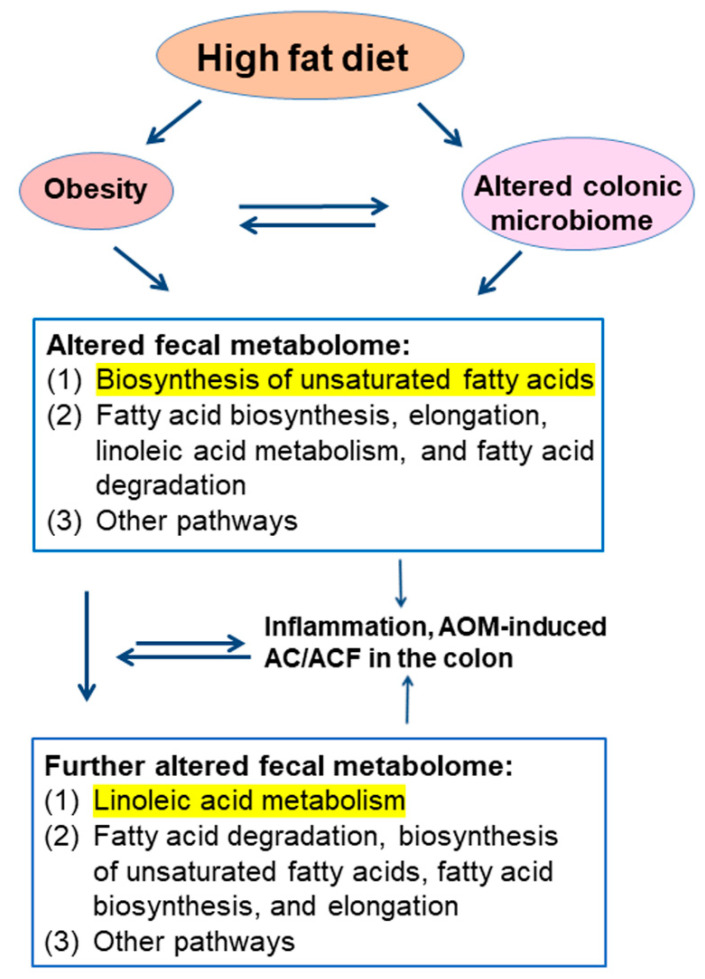
Proposed changes in top fecal metabolomic pathways in an AOM-induced colon cancer mouse model fed with a HFD. The most critical pathways are highlighted with yellow.

To explore the functional connection, we determined the interrelation of fecal (or plasma) lipid metabolites and potential oncogenic signatures in the colon. The correlation analysis demonstrated that nine fecal lipid metabolites of component 1 (Figure 1B) were positively associated with the number of AC and ACF proteins, the cell proliferation protein marker Ki67, and the abundance of dysbiotic proteobacteria (Figure 5A) [8]. Furthermore, fecal linoleic acid was also positively associated with inflammatory iNOS protein and potential dysbiotic bacteria (Tenericutes) (Figure 5A) [8]. In contrast, only the concentrations of plasma linoleic acid and oleic acid were positively associated with (AC and ACF) and (Ki67 and Proteobacteria), respectively (Figure 5B). Consistent with the fecal data (Figure 5A), the plasma lipid data (Figure 5B) further suggest the connection between linoleic acid, oleic acid, and oncogenic signatures in the colon. These data suggest several biological implications: (1) at the colonic microenvironment and systemic levels, the critical role of the metabolism of certain fatty acids (e.g., linoleic acid) in colonic inflammation and tumorigenesis is underscored in the context of diet-induced obesity; (2) compared to plasma lipid metabolites, fecal lipid metabolites are more suitable/sensitive for seeking potential colon cancer biomarkers; (3) the combination of both fecal and plasma lipid profiles will provide a new molecular basis and additional validation when seeking colon health markers. Collectively, our data suggest that the process of AOM-induced ACF formation is associated with a change in fecal metabolome, and may increase certain fecal lipid signatures correlated with the effects of gut inflammation and tumorigenesis in the colon in a male mouse model fed with a HFD (Figure 6). If this correlation also exists in our future female mouse experiments, the identification of fecal metabolome signatures in humans may open new avenues for seeking non-invasive colon health biomarkers. 

## 4. Materials and Methods 

### 4.1. Animals, Diets, and AOM Treatment

The study design, diet composition, and preparation have been previously reported [8]. Three- to four-week-old male C57BL/6 mice (Harlan, Madison, WI) were individually housed in Plexiglas^TM^ ventilated cages within a pathogen-free facility that maintained a 12 h light–dark cycle and a temperature of 22 ± 1 °C. Mice were given free access to food and deionized water. This study was approved by the Animal Care and Use Committee of the Grand Forks Human Nutrition Research Center (protocol code HZ13M2), and animals were maintained in accordance with NIH guidelines for the care and use of laboratory animals. Briefly, C57BL/6 mice were randomly assigned to either an AIN or HFD group (*n* = 25/group) for the entire experimental period (14 weeks). On week 3, within a given diet group, mice received either weekly intraperitoneal injections of the colon carcinogen, AOM (Sigma, St. Louis, MO) (*n* = 15/group) at a concentration of 8 mg/kg body weight [30], or phosphate buffered saline (PBS, pH = 7.4) carrier solution (*n* = 10/group) for 4 weeks. At the termination of the experiment, mice were fasted for 6 h and then euthanized with a mixture of ketamine and xylazine (100 mg/kg body weight). At the end of the study (week 14), (A) fecal and plasma samples were collected and stored at −80 °C for metabolomic analysis [13], and (B) ileum and colon samples were fixed for immunohistochemistry protein, AC, and ACF analyses [8].

### 4.2. Fecal Metabolomics 

Metabolomic analysis was performed at the West Coast Metabolomics Center (University of California, Davis Genomic Center, Davis, CA, USA) [31,32]. Fecal samples were extracted and derivatized by silylation and methyloximation, and analyzed by GC-TOF-MS for untargeted metabolomics with >95% accuracy for metabolite species identification. Data were processed at the West Coast Metabolomics Center using the BinBase database [33]. Metabolite quantifier ion peak heights were normalized to the sum intensities of all known compounds and used for the follow-up statistical analyses (Table S1).

### 4.3. Statistical and Bioinformatic Analysis 

To avoid a skewed distribution when performing bioinformatic analysis, the obtained (peak-intensity) data were normalized by Log10 transformation with an auto-scaling method which is highly recommended for most peak intensity data using MetaboAnalyst software (version 5.0, McGill University, Sainte Anne de Bellevue, QC, Canada) [34,35]. The metabolite group separation and functional pathways were analyzed by sPLSDA, pathway enrichment analysis, and metabolite set enrichment analysis using the MetaboAnalyst software [34,35], respectively. The effects of the relative abundance of fecal metabolites were analyzed using a two-way analysis of variance (ANOVA), corrected by a false discovery rate (FDR) of 0.05, and Tukey’s contrasts for post-hoc comparisons. Results are given as mean ± standard deviation (SD). JMP V15 (SAS Institute Inc., Cary, NC) and MetaboAnalyst software (version 5.0, McGill University, Sainte Anne de Bellevue, QC, Canada) were used for all statistical analyses [34,35]. 

## 5. Conclusions 

Taken together, our results demonstrate that the process of AOM-induced ACF formation is associated with an increase in fecal fatty acids in male mice fed with a HFD but not an AIN. The accumulation of fecal fatty acids also correlates with changes in overall metabolome compositions and may be directly related to tumorigenesis in the colon in a male mouse model. 

## Figures and Tables

**Table 1:** The effect of AOM treatment and diet on the abundance of fecal metabolites.

Metabolites	AIN	AIN + AOM	HFD	HFD + AOM
Adenine	1.00 ± 0.19 ^a^	1.02 ± 0.29 ^a^	0.89 ± 0.26 ^ab^	0.64 ± 0.27 ^b^
Adenosine-5-monophosphate	1.00 ± 0.18 ^a^	0.88 ± 0.37 ^ab^	0.58 ± 0.25 ^bc^	0.51 ± 0.26 ^c^
Alanine	1.00 ± 0.20 ^ab^	1.34 ± 0.76 ^a^	0.84 ± 0.20 ^b^	0.70 ± 0.25 ^b^
Alanine-alanine	1.00 ± 0.47 ^ab^	1.19 ± 0.57 ^a^	0.73 ± 0.24 ^ab^	0.62 ± 0.33 ^b^
Arachidic acid	1.00 ± 0.36 ^c^	0.77 ± 0.27 ^c^	3.55 ± 0.76 ^b^	4.72 ± 1.13 ^a^
Aspartic acid	1.00 ± 0.57 ^ab^	1.48 ± 0.87 ^a^	0.51 ± 0.25 ^bc^	0.34 ± 0.24 ^c^
Azelaic acid	1.00 ± 0.50 ^c^	1.23 ± 0.55 ^bc^	1.80 ± 0.78 ^ab^	2.02 ± 0.40 ^a^
Benzoic acid	1.00 ± 0.22 ^b^	0.98 ± 0.27 ^b^	1.46 ± 0.28 ^a^	1.17 ± 0.34 ^ab^
Beta-alanine	1.00 ± 0.24 ^a^	0.86 ± 0.28 ^ab^	0.64 ± 0.19 ^bc^	0.62 ± 0.17 ^c^
Beta-glutamic acid	1.00 ± 0.66 ^a^	0.94 ± 0.37 ^a^	0.63 ± 0.20 ^ab^	0.54 ± 0.23 ^b^
Biphenyl	1.00 ± 0.46 ^ab^	0.88 ± 0.39 ^b^	1.37 ± 0.28 ^a^	0.88 ± 0.31 ^b^
Cellobiose	1.00 ± 0.77 ^a^	0.71 ± 0.33 ^ab^	0.26 ± 0.10 ^b^	0.32 ± 0.29 ^b^
Cholesterol	1.00 ± 0.30 ^c^	1.09 ± 0.47 ^c^	1.58 ± 0.34 ^b^	2.24 ± 0.56 ^a^
Cis-gondoic acid	1.00 ± 0.48 ^b^	0.62 ± 0.26 ^b^	3.62 ± 1.56 ^b^	10.57 ± 6.68 ^a^
Citramalic acid	1.00 ± 0.19 ^ab^	0.88 ± 0.21 ^ab^	1.09 ± 0.28 ^a^	0.78 ± 0.18 ^b^
Citric acid	1.00 ± 0.55 ^a^	0.96 ± 0.45 ^a^	0.56 ± 0.31 ^ab^	0.36 ± 0.13 ^b^
Creatinine	1.00 ± 0.65 ^a^	0.45 ± 0.33 ^b^	0.30 ± 0.16 ^b^	0.45 ± 0.44 ^b^
Deoxycholic acid	1.00 ± 0.38 ^c^	0.72 ± 0.41 ^c^	4.64 ± 1.14 ^a^	1.90 ± 0.58 ^b^
Ethanolamine	1.00 ± 0.80 ^a^	0.73 ± 0.52 ^ab^	0.38 ± 0.19 ^b^	0.45 ± 0.31 ^b^
Fructose	1.00 ± 0.79 ^a^	0.58 ± 0.38 ^ab^	0.29 ± 0.14 ^b^	0.36 ± 0.31 ^b^
Glucose	1.00 ± 0.86 ^a^	0.56 ± 0.36 ^ab^	0.23 ± 0.24 ^b^	0.14 ± 0.12 ^b^
Glyceric acid	1.00 ± 0.37 ^ab^	1.23 ± 0.32 ^a^	0.89 ± 0.20 ^bc^	0.59 ± 0.24 ^c^
Glycerol-3-galactoside	1.00 ± 0.46 ^ab^	0.68 ± 0.23 ^c^	1.18 ± 0.28 ^a^	0.71 ± 0.20 ^bc^
Glycerol-alpha-phosphate	1.00 ± 0.31 ^ab^	1.17 ± 0.37 ^a^	0.79 ± 0.24 ^b^	0.78 ± 0.26 ^b^
Glycolic acid	1.00 ± 0.24 ^b^	0.99 ± 0.34 ^b^	1.48 ± 0.44 ^a^	0.87 ± 0.30 ^b^
Heptadecanoic acid	1.00 ± 0.42 ^b^	0.94 ± 0.34 ^ab^	1.37 ± 0.37 ^a^	1.36 ± 0.40 ^a^
Hexadecylglycerol	1.00 ± 0.71 ^b^	0.57 ± 0.31 ^b^	2.94 ± 1.31 ^b^	7.83 ± 5.34 ^a^
Isomaltose	1.00 ± 0.97 ^a^	0.47 ± 0.47 ^ab^	0.16 ± 0.03 ^b^	0.19 ± 0.05 ^b^
Isothreonic acid	1.00 ± 0.70 ^a^	0.56 ± 0.58 ^ab^	0.37 ± 0.34 ^b^	0.36 ± 0.40 ^b^
Linoleic acid	1.00 ± 0.40 ^c^	0.95 ± 0.41 ^c^	3.26 ± 0.76 ^b^	6.64 ± 2.38 ^a^
Lysine	1.00 ± 0.40 ^ab^	0.90 ± 0.36 ^b^	1.66 ± 0.76 ^a^	1.32 ± 0.67 ^ab^
Maltose	1.00 ± 0.53 ^a^	0.56 ± 0.26 ^b^	0.16 ± 0.07 ^c^	0.20 ± 0.25 ^c^
N-acetylglutamate	1.00 ± 0.65 ^a^	1.96 ± 0.92 ^b^	0.47 ± 0.31 ^b^	0.56 ± 0.26 ^b^
Nicotinic acid	1.00 ± 0.42 ^ab^	1.19 ± 0.33 ^a^	0.79 ± 0.19 ^b^	0.69 ± 0.17 ^b^
Octadecanol	1.00 ± 0.55 ^b^	0.81 ± 0.31 ^b^	1.16 ± 0.41 ^ab^	1.91 ± 1.35 ^a^
Octadecylglycerol	1.00 ± 0.53 ^b^	0.66 ± 0.31 ^b^	2.93 ± 0.98 ^b^	7.08 ± 4.28 ^a^
Oleic acid	1.00 ± 0.25 ^b^	0.92 ± 0.40 ^b^	2.71 ± 1.17 ^b^	8.47 ± 5.08 ^a^
Palmitic acid	1.00 ± 0.26 ^c^	1.00 ±0.26 ^c^	2.12 ± 0.29 ^b^	3.44 ± 1.01 ^a^
Palmitoleic acid	1.00 ± 0.53 ^b^	0.81 ± 0.37 ^b^	2.24 ± 1.03 ^b^	4.07 ± 2.17 ^a^
Phenylacetic acid	1.00 ± 0.37 ^c^	1.29 ± 0.56 ^bc^	1.74 ± 0.61 ^ab^	1.95 ± 0.66 ^a^
Pimelic acid	1.00 ± 0.26 ^b^	0.88 ± 0.29 ^b^	1.35 ± 0.29 ^a^	1.00 ± 0.21 ^b^
p-tolyl glucuronide	1.00 ± 0.73 ^b^	0.83 ± 0.95 ^b^	1.01 ± 0.72 ^b^	2.33 ± 1.79 ^a^
Putrescine	1.00 ± 0.38 ^b^	0.56 ± 0.29 ^b^	4.71 ± 5.34 ^a^	2.58 ± 2.18 ^ab^
Sophorose	1.00 ± 1.20 ^a^	0.31 ± 0.35 ^b^	0.09 ± 0.05 ^b^	0.08 ± 0.05 ^b^
Stearic acid	1.00 ± 0.24 ^c^	1.12 ± 0.42 ^c^	2.45 ± 0.63 ^b^	3.15 ± 0.89 ^a^
Stigmasterol	1.00 ± 0.33 ^b^	0.87 ± 0.42 ^b^	1.90 ± 0.99 ^a^	1.13 ± 0.86 ^b^
Tartaric acid	1.00 ± 1.20 ^a^	0.58 ± 0.74 ^ab^	0.10 ± 0.05 ^b^	0.10 ± 0.10 ^b^
Threonic acid	1.00 ± 0.67 ^a^	0.40 ± 0.31 ^b^	0.49 ± 0.37 ^b^	0.38 ± 0.31 ^b^
Thymine	1.00 ± 0.25 ^ab^	1.13 ± 0.30 ^a^	0.59 ± 0.30 ^c^	0.70 ± 0.26 ^bc^
Tocopherol beta	1.00 ± 0.29 ^c^	1.07 ± 0.47 ^c^	5.44 ± 2.24 ^a^	3.64 ± 1.80 ^b^
Triethanolamine	1.00 ± 0.28 ^ab^	0.86 ± 0.32 ^b^	1.28 ± 0.54 ^a^	0.85 ± 0.24 ^b^
Tyramine	1.00 ± 0.66 ^b^	1.77 ± 1.16 ^ab^	2.82 ± 1.56 ^a^	1.79 ± 1.16 ^ab^
UDP-N-acetylglucosamine	1.00 ± 0.39 ^b^	1.47 ± 0.50 ^a^	1.06 ± 0.36 ^ab^	0.88 ± 0.38 ^b^
Uracil	1.00 ± 0.32 ^ab^	1.14 ± 0.30 ^a^	0.71 ± 0.22 ^b^	0.78 ± 0.23 ^b^
Urocanic acid	1.00 ± 0.28 ^a^	0.86 ± 0.23 ^ab^	0.81 ± 0.25 ^ab^	0.70 ± 0.13 ^b^
1,3-Diaminopropane	1.00 ± 0.42 ^bc^	0.68 ± 0.21 ^c^	3.20 ± 2.42 ^a^	1.99 ± 1.28 ^ab^
1-Hexadecanol	1.00 ± 0.72 ^b^	1.04 ± 0.82 ^b^	1.15 ± 0.82 ^ab^	2.71 ± 2.46 ^a^
1-Monoolein	1.00 ± 0.57 ^a^	0.71 ± 0.38 ^ab^	0.39 ± 0.12 ^b^	0.40 ± 0.16 ^b^
2-Hydroxyvaleric acid	1.00 ± 0.20 ^b^	1.11 ± 0.40 ^ab^	1.45 ± 0.34 ^a^	1.25 ± 0.27 ^ab^
2-Methylglyceric acid	1.00 ± 0.27 ^b^	1.01 ± 0.37 ^b^	1.43 ± 0.31 ^a^	1.08 ± 0.27 ^b^
3-Epicholic acid	1.00 ± 1.32 ^ab^	0.53 ± 0.55 ^b^	2.20 ± 2.41 ^a^	0.47 ± 0.36 ^b^
3-Hydroxy-3-methyglutaric acid	1.00 ± 0.26 ^ab^	0.89 ± 0.46 ^b^	1.45 ± 0.57 ^a^	1.02 ± 0.36 ^ab^
3-Phenyllactic acid	1.00 ± 0.35 ^ab^	0.98 ± 0.60 ^a^	0.55 ± 0.23 ^bc^	0.48 ± 0.18 ^c^
4-Aminobutyric acid	1.00 ± 0.26 ^b^	0.89 ± 0.22 ^b^	1.63 ± 1.00 ^a^	0.83 ± 0.25 ^b^
6-Hydroxynicotinic acid	1.00 ± 0.30 ^b^	0.96 ± 0.40 ^b^	2.34 ± 0.40 ^a^	1.14 ± 0.38 ^b^

Values are means ± SDs, *n* = 10/group (AIN or HFD group) and *n* = 15/group (AIN + AOM or HFD + AOM group). Data from the HFD, AIN + AOM, and HFD + AOM groups were converted to fold changes compared to the AIN group. For a given metabolite, if two values do not share at least one common letter (a, b, or c), then the difference between them is statistically significant (*p* < 0.05 is adjusted by the FDR method).

**Table 2:** (A): In the AIN vs HFD group, the top 8 significant metabolic pathways are involved in the 65 altered metabolites *. (B): In the HFD vs. HFD + AOM group, the top 8 significant metabolic pathways are involved in the 65 altered metabolites **.

**A**				
**KEGG Pathway**	**Total** **Compounds**	**Hits**	**FDR *p***	Impact ***
Biosynthesis of unsaturated fatty acids	36	5	2.10 × 10^−8^	0.00
Fatty acid biosynthesis	47	1	1.46 × 10^−6^	0.01
Fatty acid elongation	39	1	1.46 × 10^−6^	0.00
Linoleic acid metabolism	5	1	1.46 × 10^−6^	1.00
Fatty acid degradation	39	2	4.93 × 10^−5^	0.00
Beta-alanine metabolism	21	4	3.54 × 10^−4^	0.40
Primary bile acid biosynthesis	46	1	2.54 × 10^−3^	0.03
Steroid biosynthesis	42	1	2.54 × 10^−3^	0.03
**B**				
**KEGG Pathway**	**Total** **Compounds**	**Hits**	**FDR *p***	Impact ***
Linoleic acid metabolism	5	1	2.48 × 10^−4^	1.00
Fatty acid degradation	39	2	3.75 × 10^−4^	0.00
Biosynthesis of unsaturated fatty acids	36	5	3.75 × 10^−4^	0.00
Fatty acid biosynthesis	47	1	5.73 × 10^−3^	0.01
Fatty acid elongation	39	1	5.73 × 10^−3^	0.00
Butanoate metabolism	15	1	7.03 × 10^−3^	0.03
Alanine, aspartate, and glutamate metabolism	28	4	7.13 × 10^−3^	0.31
Glyoxylate and dicarboxylate metabolism	32	2	7.13 × 10^−3^	0.11

* Only the top 8 of 23 statistically significant pathways (Table S3) are listed here. ** Only the top 8 of 15 statistically significant pathways (Table S4) are listed here; the *p*-values are obtained by pathway enrichment analysis and adjusted by both Holm and FDR methods. *** The pathway impact score is obtained by pathway topology analysis.

## Data Availability

All fecal metabolomics data used in this report are available as a Supplementary excel file (Table S1).

## Supplementary Materials

The following supporting information can be downloaded at: https://www.mdpi.com/article/10.3390/biomedicines10112891/s1. Table S1: Metabolites identified in fecal from C57BL/6 mice fed with the AIN or the HFD and with or without AOM treatment. Table S2: The 32 statistically significant metabolic pathways involved in the 65 altered metabolites. Table S3: In the AIN vs HFD group, the 23 statistically significant metabolic pathways are involved in the 65 altered metabolites. Table S4: In the HFD vs HFD + AOM group, the 15 statistically significant metabolic pathways are involved in the 65 altered metabolites. Table S5: The 27 significant metabolite sets are involved in the 65 altered metabolites.

## Abbreviations

AC: aberrant crypt; ACF, aberrant crypt foci; AIN, a modified AIN93G formulation; ANOVA, analysis of variance; AOM, azoxymethane; FDR, false discovery rate; GC-TOFMS; gas chromatography time-of-flight mass spectrometry; iNOS, inducible nitric oxide synthase; KEGG, Kyoto Encyclopedia of Genes and Genome; sPLSDA, sparse partial least squares discriminant analysis; 2D, two-dimensional.
